# Bibliometric Analysis of Weather Radar Research from 1945 to 2024: Formations, Developments, and Trends

**DOI:** 10.3390/s24113531

**Published:** 2024-05-30

**Authors:** Yin Liu

**Affiliations:** 1 Jiangsu Meteorological Observation Center, Nanjing 210041, China; liuyin200421@163.com; 2 College of Atmospheric Sounding, Chengdu University of Information Technology, Chengdu 610225, China; 3 Key Laboratory of Atmosphere Sounding, China Meteorological Administration, Chengdu 610225, China; 4 Key Laboratory of Transportation Meteorology, China Meteorological Administration, Nanjing 210041, China

**Keywords:** weather radar, scientometric mapping, knowledge framework, future trend

## Abstract

In the development of meteorological detection technology and services, weather radar undoubtedly plays a pivotal role, especially in the monitoring and early warning of severe convective weather events, where it serves an irreplaceable function. This research delves into the landscape of weather radar research from 1945 to 2024, employing scientometric methods to investigate 13,981 publications from the Web of Science (WoS) core collection database. This study aims to unravel, for the first time, the foundational structures shaping the knowledge domain of weather radar over an 80-year period, exploring general features, collaboration, co-citation, and keyword co-occurrence. Key findings reveal a significant surge in both publications and citations post-1990, peaking in 2022 with 1083 publications and 13832 citations, signaling sustained growth and interest in the field after a period of stagnation. The United States, China, and European countries emerge as key drivers of weather radar research, with robust international collaboration playing a pivotal role in the field’s rapid evolution. Analysis uncovers 30 distinct co-citation clusters, showcasing the progression of weather radar knowledge structures. Notably, deep learning emerges as a dynamic cluster, garnering attention and yielding substantial outcomes in contemporary research efforts. Over eight decades, the focus of weather radar investigations has transitioned from hardware and software enhancements to Artificial Intelligence (AI) technology integration and multifunctional applications across diverse scenarios. This study identifies four key areas for future research: leveraging AI technology, advancing all-weather observation techniques, enhancing system refinement, and fostering networked collaborative observation technologies. This research endeavors to support academics by offering an in-depth comprehension of the progression of weather radar research. The findings can be a valuable resource for scholars in efficiently locating pertinent publications and journals. Furthermore, policymakers can rely on the insights gleaned from this study as a well-organized reference point.

## 1. Introduction

Weather radar technology emerged alongside military radar technology during World War II. In 1940, the British observed interference in radar signals when monitoring aircraft, leading to research on the impact of clouds and precipitation on these signals [1]. The findings from this research were applied during World War II, greatly aiding in the detection of hazardous weather conditions such as thunderstorms along aircraft routes. In April 1944, the United States installed weather surveillance radars at two ports in Panama facing the Atlantic, marking the birth of the world’s first “weather radar network”. In 1946, the U.S. Weather Bureau obtained 25 AN/AP2F military radars from the Navy and modified them for meteorological surveillance, including models such as WSR-1s, -1As, -3s, and -4s, which were predecessors to the later WSR series weather radars [2]. Subsequent developments led to the WSR-57, WSR-74C, WSR-74S, and other radar models with different functions and features, such as AN/APQ-13, AN/CPS-9, and AN/FPS-103, culminating in the present-day operational use of the WSR-88D radar [3].

Monitoring and early warning of severe weather systems are crucial factors driving the advancement and business development of weather radar technology. In the 1950s, the U.S. aviation industry entered a rapid expansion phase with the operation of large hub airports and jet aircraft, leading to an increase in major air disasters caused by weather-related issues. In response, the United States initiated the National Severe Storms Project (NSSP) in 1961 to study such catastrophic events triggered by severe weather [4]. The headquarters were established in Kansas City, Missouri, and in 1962, a research weather monitoring radar (WSR-57) was installed in Norman, Oklahoma. Notably, the WSR-57 served as the flagship radar for the meteorological bureau for a long time until the last WSR-57 was retired in December 1996 in South Carolina after the deployment of Next-Generation Radar (NEXRAD) in the 1990s [5]. In 1963, the entire NSSP was moved to Norman, where it was reorganized and transformed into the National Severe Storms Laboratory (NSSL). Following its establishment, NSSL focused on the development of Doppler weather radars. By 1964, through the modification of naval radars, the first 3 cm pulse Doppler radar was created for research on precipitation physics, storm internal flow fields, and clear-air echo variations, eventually retiring in 1970 [6]. In the late 1960s, NSSL collaborated with Cornell University to conduct the first dual-Doppler radar detection experiments. Simultaneously, in order to reduce signal attenuation and enhance Doppler radar electromagnetic wave penetration through severe storms, plans were made to develop a 10 cm radar, which was successfully implemented in 1967 after some challenges [7]. In the spring of 1971, the 10 cm Doppler radar on the campus of the University of Oklahoma was officially put into use for detection, becoming one of the few 10 cm wavelength Doppler radars globally [8]. Over the following years, it captured numerous occurrences and developments of severe storms and tornado weather systems, such as a tornado that struck Union City, Oklahoma, on 24 May 1973 [9]. The Storm Chasers team tracked and photographed the tornado formation, comparing it with continuous data detected by the Doppler radar, analyzing the complete temporal and spatial characteristics and spiral structure evolution process during the tornado event. The results of various experiments completely dispelled any doubts regarding the benefits of investing in this advanced equipment, paving the way for further advancement in weather radar technology [10,11,12,13].

In order to comprehensively enhance the monitoring and early warning capabilities for severe weather, the National Weather Service (NWS), the Air Weather Service (AWS) of the U.S. Air Force, and the Federal Aviation Administration (FAA) jointly formulated a Joint Doppler Operational Project (JDOP) [14,15]. This project was based on the implementation of NSSL’s 10 cm Doppler radar during the peak tornado season in Norman, Oklahoma, in the spring of 1977–1978. The results demonstrate a notable enhancement in the accuracy of forecasting strong storms and tornadoes, accompanied by a reduction in false alarms, and an increase in lead time from 2 min to 21 min [16,17]. Due to the resounding success of JDOP, the Doppler weather radar development project gained recognition from the U.S. government, leading to the nationwide construction of the NEXRAD Doppler weather radar network [18]. An important feature of the new radar system was its complete automation, eliminating the need for manual hardware operation. The radar automatically generated continuous three-dimensional detection data, which were then processed by software to produce various application products [19]. Operational personnel only needed to operate and display various data products on the Primary User Processor (PUP) computer terminal to identify potential weather impacts [20]. Clearly, this software processing system, incorporating a variety of complex algorithms, fully showcased the advantages of the new-generation radar and became a key factor in the successful application of NEXRAD in operational settings [21,22,23]. In 1990, the first WSR-88D, a new-generation 10 cm Doppler weather radar installed in Norman, commenced operations [24]. By 1996, over 160 units had been deployed nationwide, establishing it as the largest modern weather radar network in the world at that time [25]. It was also regarded as one of the most effective projects for meteorological business development in the United States during the 1990s.

While the United States was developing the NEXRAD network, China also formulated a plan for the development of a new generation of weather radars in 1994 [26]. Drawing lessons from the U.S. development experience, China planned to deploy 126 new-generation radars nationwide [27]. In practical terms, China decided to introduce the latest WSR-88D technology from the United States and collaborate to produce a new generation of weather radars, known as China Weather Radar Network (CINRAD) [28]. In September 1999, the first CINRAD-SA model, a new-generation weather radar produced in China, was completed in Hefei, Anhui Province, symbolizing the comprehensive launch of China’s new-generation weather radar network construction [29,30]. The construction of China’s new-generation weather radar has continued to this day, with the number of radars in the network exceeding 270, far surpassing the initial plan [31]. Together with the United States, China has formed the world’s two largest new-generation weather radar networks.

Considering the frequent occurrence of catastrophic weather globally, countries worldwide are actively advancing their respective weather radar networks. Presently, weather radar technology is advancing towards multispectrum techniques (acoustic wave–optical wave–L band–X-C-S band–Ka-W band), multiplatform technology (space–ground–air), refined detection (high precision and high spatiotemporal resolution), all-weather process observation technology (clear-sky atmospheric phase, cloud formation phase, precipitation phase, and meteorological disaster phase), and networked collaborative observation technology (multiband multisystem weather radar) [32,33,34,35,36,37,38,39,40,41,42,43,44,45,46,47,48,49]. Over the past 80 years since the introduction of the first weather radar, scholars from various countries have published tens of thousands of scientific and technological papers in the field of weather radar, making indelible contributions to the rapid development of weather radar technology. These scientific papers encompass a variety of types such as articles, reviews, letters, notes, and proceeding papers, covering all aspects of weather radar. Among these, some reviews have embraced a variety of viewpoints to address the interdisciplinary nature of weather radar research [50,51,52,53,54]. These viewpoints include hardware development, software development, signal processing, data quality control, and product application of weather radar [55,56,57,58,59]. Nevertheless, the current reviews in this field heavily lean on expert perspectives and often zero in on specific themes, thus overlooking the quantitative bibliometric analysis of the entire research domain. Even with the growing volume of literature on weather radar in the last 80 years, our grasp of the wider knowledge domain remains constrained.

In recent years, emerging bibliometric methods have provided an objective quantitative means to comprehensively analyze the development of a research field [60,61]. These methodologies leverage citation analysis within scholarly literature to delineate the knowledge structure and predict upcoming trends. To support such analyses, researchers frequently utilize visualization software such as CiteSpace [62], VOSviewer [63], and Histcite [64], which assist in exploring, extracting, analyzing, and illustrating knowledge within the scientometric sphere.

Despite the widespread application of knowledge domain mapping, as far as we know, there has been a lack of investigations exploring the emerging literature on weather radar. Recognizing the importance of acquiring a comprehensive insight into this field, it is crucial to undertake a structured investigation that provides valuable insights into the current status and potential future developments. This research endeavors to fill this void for the first time through a detailed examination of studies in the weather radar domain. By leveraging bibliographic data from the Web of Science (WoS) core collection database covering the period from 1900 to the present [65], our goal is to explore the fundamental framework of the weather radar knowledge domain and offer trustworthy forecasts for future research directions. This effort not only provides scholars with a comprehensive understanding of the knowledge landscape in the field but also offers policymakers a well-defined benchmark.

The organization of this paper is as follows: Section 2 contains information on data preprocessing, analysis tool, and technical roadmap. The findings of the analysis are outlined in Section 3, with Section 4 focusing on possible future research avenues derived from the insights gained. Finally, we summarize the main discoveries and demonstrate the extensive significance and applicability of our research.

## 2. Materials and Methods

### 2.1. Data Gathering and Preprocessing

This research utilized data obtained from the WoS core collection database spanning from 1900 to the present. The search was conducted on 22 February 2024, revealing that the earliest publication related to weather radar dates back to 1945. Consequently, the study’s literature collection timeframe was defined as 1 January 1945 to 22 February 2024. By using the search term “TS = (weather radar OR precipitation radar OR atmospheric radar OR Doppler radar)”, a comprehensive set of 18,641 publications was identified. The search results were formatted as “Full Record and Cited References” in plain text. After excluding conference papers, meeting abstracts, editorials, notes, corrections, and book chapters, a dataset comprising 13,981 publications consisting of review papers and original research articles was compiled.

### 2.2. Analysis Tool

CiteSpace is a popular software tool employed for conducting bibliometric analysis and visualizing the evolution of research areas, along with the associations among keywords, authors, and institutions [66]. Its main purpose is to analyze extensive amounts of publication details obtained from academic databases. CiteSpace enables users to create a variety of visual representations that include popular papers, author networks, and keyword clusters. These visualizations offer valuable perspectives on the evolution of academic areas, spotlighting significant academic contributions and noteworthy researchers, while revealing developing patterns and potential directions for future study [67].

In this study, we utilized an advanced version of CiteSpace 6.3.R1 as a vital tool for managing publications obtained from the WoS database and creating interactive graphics. The utilization of CiteSpace proved instrumental in facilitating researchers’ comprehensive understanding of the weather radar domain.

### 2.3. Technical Roadmap

**Figure 1:**
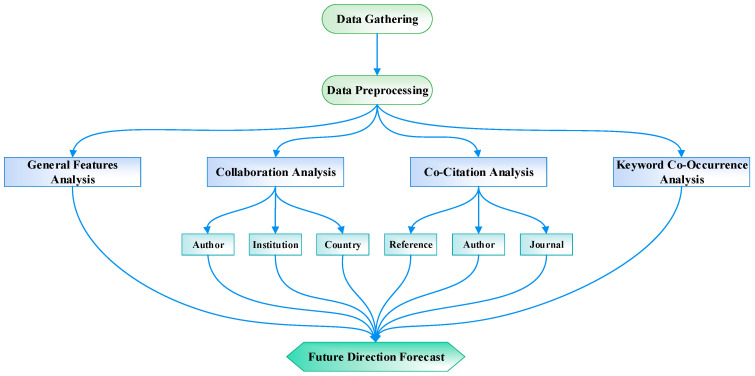
Technical roadmap.

Figure 1 illustrates the technical roadmap of this study. The historical literature on weather radar is initially gathered and preprocessed, followed by analyses from four distinct perspectives: general features, collaboration, co-citation, and keyword co-occurrence. The collaborative analysis explores possible partnerships, encompassing analyses across authors, institutions, and countries. The co-citation analysis aims to reveal the basic information structuring, involving analyses of references, authors, and journals. Meanwhile, keyword co-occurrence analysis is employed to monitor the evolution of research trends and identify current focal points. Furthermore, the keyword co-occurrence analysis is employed to observe the evolution of research themes and pinpoint noteworthy topics. Finally, based on the conclusions drawn from the analyses, reasonable and credible predictions are made regarding the future development direction of the weather radar field.

## 3. Results

### 3.1. General Features

**Figure 2:**
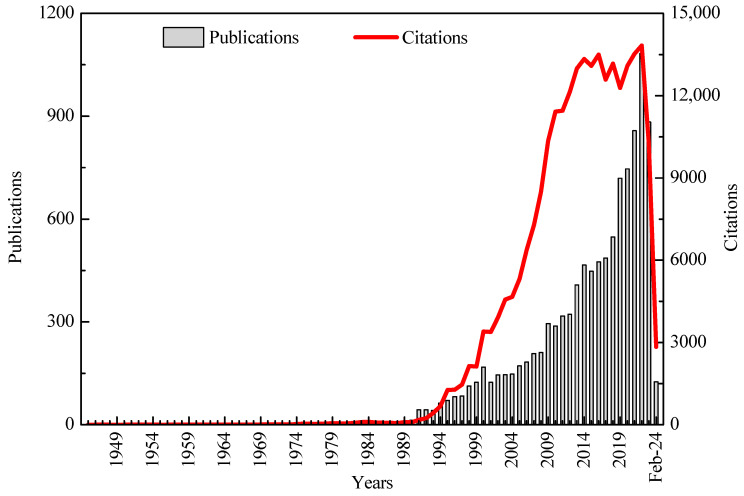
The annual number of publications and citations within the field of weather radar research.

Figure 2 presents the annual number of publications and citations within the field of weather radar research. It can be observed that from 1945 to 1990, the number of papers published and citations in the field of weather radar research was very limited, indicating a period of stagnation in the development of this research field. Subsequent analysis indicates that the main reason is that during this period, weather radar research was just getting started and did not receive much attention, with a very small number of researchers in the field. However, from 1990 to the present, the number of publications and citations in the field of weather radar research has been increasing rapidly year by year, signaling a period of rapid development in this research domain. Notably, the peak occurred in 2022, with 1083 publications and 13832 citations. Additionally, the yearly citation count in the realm of weather radar research shows an ongoing steep incline, reflecting a sustained uptick in interest and focus on this particular field. It should be noted that the WoS core collection database did not include abstracts before 1990. This means that the articles referenced in this study prior to 1990 were primarily identified through title and author keywords. Considering the consistent year-on-year increase in the number of publications from 1945 to 2024, the overall impact on the article statistics in this study is relatively minimal.

**Figure 3:**
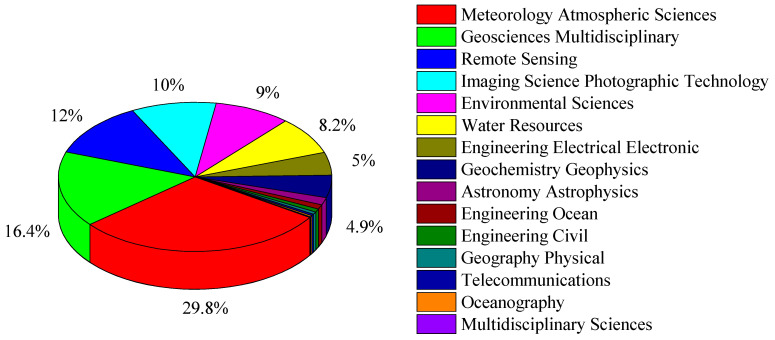
The distribution of WoS categories across 13981 articles in this research.

Figure 3 showcases the distribution of WoS categories across 13981 articles in this research. The statistical findings highlight the broad coverage of WoS categories within the field of weather radar research. Notably, the top six WoS categories are “Meteorology Atmospheric Sciences”, “Geosciences Multidisciplinary”, “Remote Sensing”, “Imaging Science Photographic Technology”, “Environmental Sciences”, and “Water Resources”. These categories encompass 4168, 2296, 1682, 1404, 1263, and 1141 articles, respectively, representing proportions of 29.8%, 16.4%, 12.0%, 10.0%, 9.0%, and 8.2% of the total dataset.

### 3.2. Collaboration Analysis

Utilizing collaborative analysis proves to be an effective approach in uncovering the intricate scientific network, facilitating the identification of key researchers at different tiers including national connections, institutional ties, and individual author contributions [62].

#### 3.2.1. Author Collaboration

**Figure 4:**
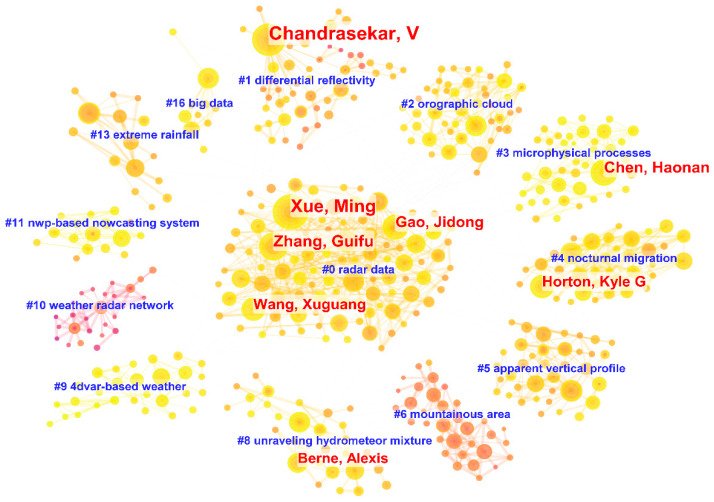
Author collaboration analysis: a circular view (Appendix A.6).

Figure 4 provides a visual illustration of the elaborate web of author collaborations, encompassing 1857 individual nodes (Appendix A.1 and Appendix A.2) and 2346 interconnected links (Appendix A.3) dating from 1945 to 2024. The nodes represent various authors, while the links indicate the cooperative connections forged between them. The blue text in the figure represents cluster (Appendix A.4) labels, while the red text represents authors with a high ranking in terms of the number of published papers. It can be noted that the research branch of “radar data” has garnered the highest level of attention and has given rise to a relatively large number of prolific authors. Table 1 presents the top 10 authors in the field of weather radar research, ranking them by the number of published articles, along with their betweenness centrality (Appendix A.5) and the earliest publication date. Standing out as a prominent researcher in the Department of Electrical and Computer Engineering at Colorado State University, Chandrasekar V. has emerged as a leading figure in the field of weather radar research, boasting an impressive publication record of 118 articles. Notably, Chandrasekar V. holds the highest centrality score of 0.13 and initiated publications in this area as early as 1992. This implies that Chandrasekar V. is an author of great significance in the field of weather radar research, and his publications have had a profound and far-reaching impact on the development of this particular area of study. A noteworthy young scholar, Chen Haonan, hailing from Colorado State University, has amassed a total of 48 publications on weather radar since his initial article in 2015. Consequently, his publication count swiftly escalated to fourth place. Simultaneously, his centrality has reached a high of 0.05, indicating a relatively strong academic influence. Researchers aiming to delve deeper into the current research focal points and emerging trends in the weather radar field should pay particular attention to the articles authored by Chen Haonan and his research team.

#### 3.2.2. Institution Collaboration

**Figure 5:**
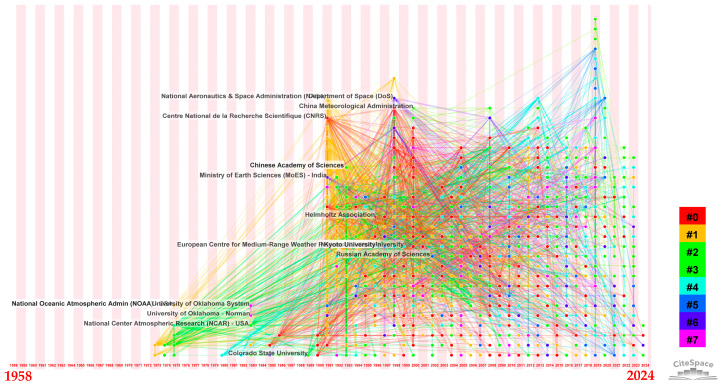
Institution collaboration analysis: a timezone view (Appendix A.7).

The visual depiction in Figure 5 illustrates the formation of academic collaborations within a network consisting of 894 nodes and 6346 links. The nodes represent various institutions, while the links indicate the cooperative connections forged between them. It is evident that institutional collaboration has been progressively widening since 1973, with a peak in the level of cooperation between institutions worldwide after 1991, which has continued until today. This phenomenon is closely intertwined with the profound changes in communication methods and modes of travel brought about by socioeconomic development. Moreover, Figure 5 also demonstrates that extensive and in-depth academic collaboration among institutions globally is a crucial factor enabling the rapid advancement of the weather radar research area. Table 2 presents a collection of the top 10 institutions that have made notable achievements in academic papers. Within the highest-ranking academic institutions, excluding one institution from France, the remaining institutions exclusively hail from the United States and China. This occurrence underscores the absolute dominance of American and Chinese institutions in the realm of weather radar research, a correlation directly influenced by the fact that these two nations boast the world’s two largest weather radar networks.

#### 3.2.3. Country Collaboration

**Figure 6:**
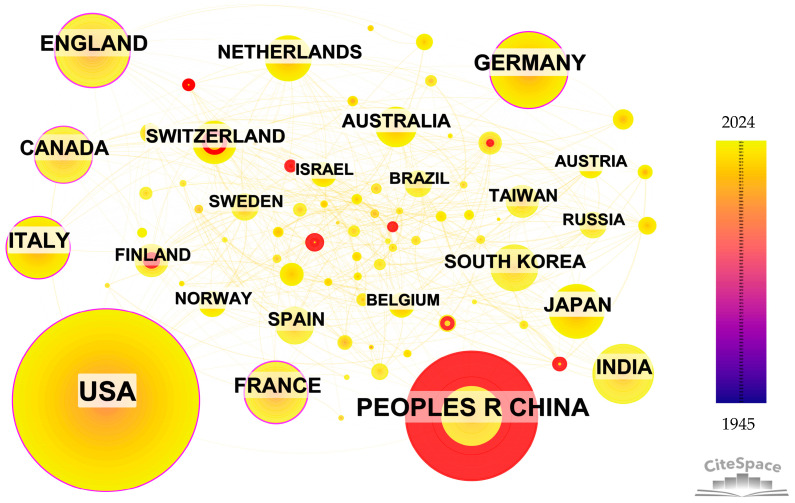
Country collaboration analysis.

Figure 6 displays the network of inter-country cooperation relationships from 1945 to 2024, comprising 126 nodes and 1123 links. The different colors of the nodes correspond to various publication times, while the links between nodes represent collaborations among different countries. Furthermore, Table 3 presents the leading 10 countries in relation to both the volume of publications and betweenness centralities. According to Figure 6 and Table 3, it is evident that the USA leads with the highest number of publications at 4926, significantly surpassing other countries. China, Germany, and England closely follow with article numbers of 2893, 923, and 895, respectively. It is noteworthy that despite the USA having the highest number of publications, its betweenness centrality is not the highest. England attains the betweenness centrality peaks at 0.26, whereas its publication count only ranks fourth. This implies that there is no evident strong connection between publication quantity and betweenness centrality. Scholars undertaking a literature analysis should be mindful of this aspect. Overall, developed countries exhibit a higher standard of research achievement in the field of weather radar compared with developing nations, as indicated by their superior publication volume and betweenness centrality metrics. Furthermore, the purple circles in Figure 6 represent high betweenness centrality, while the red circles indicate high burstness. Therefore, the USA, Germany, England, Italy, and Canada serve as crucial gateways connecting two distinct branches in the field of weather radar research, whereas China stands out as the most active and concentrated hub for emerging trends in weather radar research.

### 3.3. Co-Citation Analysis

Co-citation analysis is commonly divided into three groups: reference, author, and journal [62]. More details can be found in Appendix A.8.

#### 3.3.1. Reference Co-Citation

**Figure 7:**
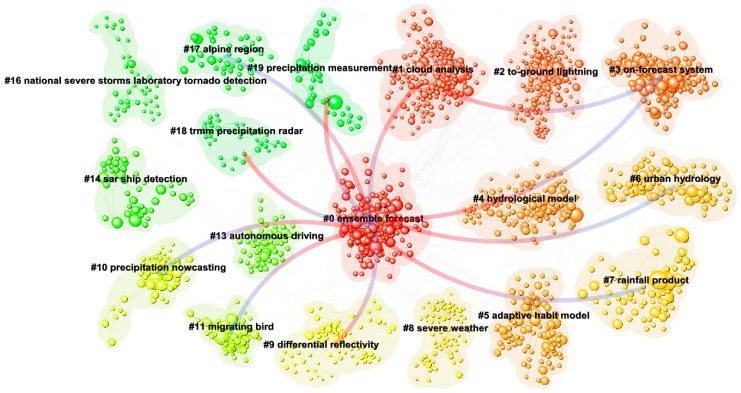
Reference co-citation analysis.

This research utilizes a log-likelihood ratio (LLR) weighting algorithm to evaluate publications and the cited references, helping assign precise professional labels for accurate cluster identification and categorization [67]. Figure 7 showcases that the co-citation network includes 2856 nodes and 9547 links, as depicted visually. This network can be divided into 30 unique co-citation clusters, with detailed information on the top 18 clusters available in Table 4 (excluding irrelevant clusters). It is worth highlighting that all clusters obtained an impressive silhouette score (Appendix A.9), indicating a robust level of coherence within the co-citation network. The designations given to the clusters in Table 4 signify long-standing and persistent research labels within the field of weather radar. Therefore, to uncover the knowledge framework of this field, a comprehensive investigation and scrutiny of the clusters are essential. This study selects the top three clusters based on their size as examples and conducts detailed analyses on them.

(1) Ensemble forecast

The largest group of individuals within the cluster, referred to as the “Ensemble forecast”, consists of 168 members and exhibits a silhouette value of 0.889. The primary referenced publication within the cluster is “Assimilation of zdr columns for improving the spinup and forecast of convective storms in storm-scale models: proof-of-concept experiments” authored by Carlin J. T., which was published in the journal *Monthly Weather Review* in 2017 [68]. The member with the highest number of citations in this cluster is “Short-wavelength technology and the potential for distributed networks of small radar systems” by McLaughlin D., which was published in the journal *Bulletin of the American Meteorological Society* in 2009 [69] and has been cited a total of 50 times.

(2) Cloud analysis

The second significant cluster, named “Cloud analysis”, is composed of 160 members and demonstrates a silhouette value of 0.933. The primary referenced publication within the cluster is “Review of the different sources of uncertainty in single polarization radar-based estimates of rainfall” by Villarini G., which was published in the journal *Surveys in Geophysics* in 2010 [70]. The member with the highest number of citations in this cluster is “A Description of the Advanced Research WRF Model Version 4” by Skamarock W. C., which was published in *NCAR Tech. Note* in 2019 [71] and has been cited a total of 92 times.

(3) To-ground lightning

The cluster associated with “To-ground lightning” ranks as the third most extensive, comprising 132 members and attaining a silhouette value of 0.947. The primary referenced publication within the cluster is “Radar hydrology: rainfall estimation” authored by Krajewski W. F., which was published in the journal *Advances in Water Resources* in 2002 [72]. The member with the highest number of citations in this cluster is “The WSR-88D rainfall algorithm” by Fulton R. A., which was published in the journal *Weather and forecasting* in 1998 [73] and has been cited a total of 37 times.

#### 3.3.2. Author Co-Citation

**Figure 8:**
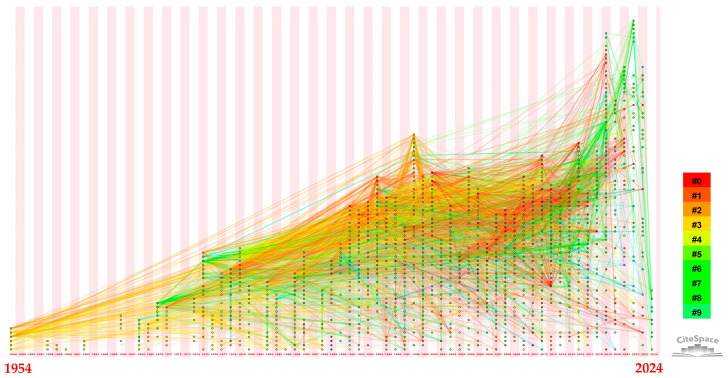
Author co-citation analysis: a timezone view.

The primary goal of author co-citation analysis is to recognize highly cited scholars and evaluate the thematic breadth of their publications in the field of weather radar. Drawing from the data showcased in Figure 8, a consolidated network of author co-citation is formulated, encompassing 1943 nodes and 12462 links. Within this interconnected system, every node signifies a distinct author, and the links reflect the relationships of co-citation among them. It can be observed that the number of highly influential authors in the field of weather radar has shown a tendency for annual increase. Simultaneously, research in this field over the past 80 years has demonstrated strong continuity and heritage. Furthermore, Table 5 presents the ranking of the top 10 authors based on their co-citation frequency, centrality, and burst strength (Appendix A.10). For emerging researchers keen on the realm of weather radar research, consulting Table 5 enables them to promptly pinpoint extensively referenced authors, pivotal thought leaders, and investigators engaged in pioneering inquiries. This, in turn, empowers them to selectively engage in ongoing tracking and observation.

#### 3.3.3. Journal Co-Citation

**Figure 9:**
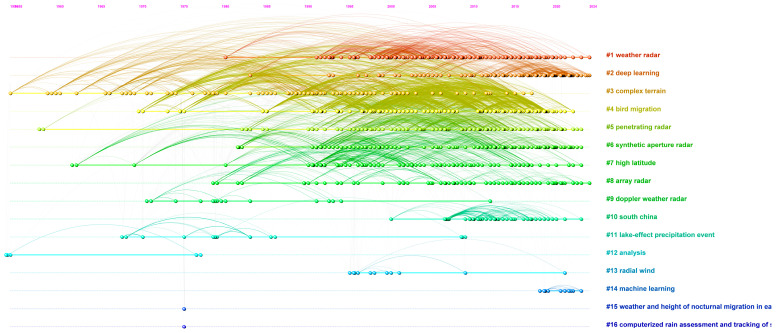
Journal co-citation analysis: a timeline view (Appendix A.11).

**Figure 10:**
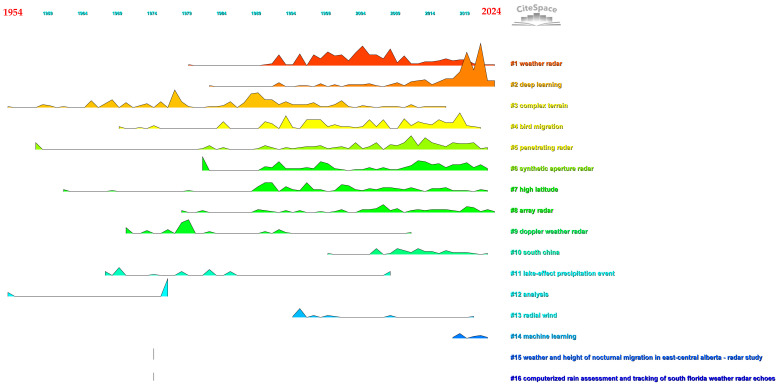
Journal co-citation analysis: a landscape view (Appendix A.12).

Similar to author co-citation analysis, the main objective of journal co-citation analysis is to identify frequently cited journals and evaluate their thematic contributions within the field of weather radar research. The creation of a unified network depicting co-citation interactions among journals, as depicted in Figure 9, comprises 2136 nodes and 13,452 links that symbolize co-citation relationships. In this network, each journal is represented as a node, with the links between them indicating the co-citation associations. The findings suggest that the initial journals within the domain of weather radar studies have continuously formed co-citation connections with subsequent relevant journals. This trend is especially apparent in the top ten clusters, signifying the thematic continuity and overlap of research content across time for journals related to weather radar. Figure 10 provides an overview of the results of the journal co-citation analysis from a landscape perspective. Each cluster is represented with the following details: its inception, duration, and conclusion, or whether it remains active. The peak’s height reflects the number of cluster members published in that specific year, serving as an indicator of the cluster’s level of activity. The results indicate that deep learning is the most active cluster in the current research field of weather radar, receiving the highest attention and yielding the most significant outcomes. To provide a clearer insight into the analysis results, Table 6 displays the ranking of the leading 10 journals according to the co-citation frequency, centrality, and burst strength. Researchers, whether in the preparation phase or actively involved in weather radar research, can discover suggestions tailored to them in Table 6, including top-cited journals, pivotal influential journals, and leading-edge thematic journals.

### 3.4. Keyword Co-Occurrence

To remain current with the latest advancements and primary research areas in the field of weather radar, an investigation into keyword co-occurrence (Appendix A.8) was undertaken. The results of this analysis are presented in Table 7, depicting a complex network of associated keywords with 1178 nodes and 9264 links. Within Table 7 (exclude irrelevant keywords), the scope of keyword presence within the weather radar domain is highlighted through bold lines, while the significance or occurrence of keyword bursts is denoted by the application of red coloring. It can be observed that the early research focus in the field of weather radar was primarily on the research and improvement of weather radar hardware and software (such as Doppler radar, WSR 88D, sensitivity, range, reflectivity, and retrieval), as well as the initial applications of observational products (mesoscale, boundary layer, fields, propagation, rainfall, hydrology, shape). In recent years, the research spotlight in this field has significantly shifted towards the integration of Artificial Intelligence (AI) technologies (machine learning, deep learning, convolutional neural network) and multifunctional applications across multiple scenarios (upgrade radar images, numerical weather prediction/forecasting, laser radar, scanning strategy, fine feature extraction, networked radars, observation mode, refined detection, collaborative observation). Particularly, the burst strength of the keyword “deep learning” has reached its peak value of 52.86, indicating that this is currently the most attention-grabbing research hotspot and the most important future research direction in the field of weather radar. It is worth noting that this conclusion is consistent with the conclusion drawn in Figure 10.

## 4. Prospective Areas for Future Research

Over the course of the last 80 years, substantial progress has been achieved in delving into the domain of weather radar research. As our comprehension evolves, it is certain that new pathways and approaches will come to light. Therefore, based on the insights shared in Section 3, we delineate the subsequent areas deserving of investigation in upcoming studies.

### 4.1. The Application of AI Technology in the Field of Weather Radar Research (Keywords in Table 7: Machine Learning, Deep Learning, Convolutional Neural Network)

In recent years, with the continuous improvement of AI technology and computational power, deep learning technology has made significant progress in many challenging tasks such as machine translation, image processing, and autonomous driving, achieving historically exceptional results [74,75]. In the era of rapid development of AI technology, countries around the world have also been conducting research on the deep integration of AI, big data, quantum computing, and meteorology applications. AI is bringing new opportunities to meteorological science, especially in the acquisition and utilization of global and regional meteorological data, as well as the prediction and forecasting of extreme weather and disasters, playing an increasingly important role with very broad application prospects. For example, in September 2021, Google DeepMind and the University of Reading in the UK jointly published an operational short-term rainfall prediction model in *Nature*, which can predict rainfall probability for the next 90 min based on radar observations from the past 20 min, with the ability to forecast rainfall amount, time, and location up to 2 h in advance, attracting high attention from meteorological service departments and relevant scholars [76].

Currently, the research field of weather radar is gradually exploring the use of deep learning models to optimize the quality of weather radar data [77,78,79]. However, due to its heavy reliance on short-term extrapolation methods based on large radar datasets, the accuracy of its extrapolations remains at a relatively low level [80,81]. In the future, emphasis can be placed on the following three aspects for related research: (1) Developing radar image quality control methods based on convolutional encoding–decoding networks to leverage the advantage of deep learning in automatically extracting features from massive data. This approach aims to repair missing data caused by radial beam blockage in weather radar, thereby enhancing radar data quality and providing higher-quality training data for short-term extrapolation models of weather radar. (2) Leveraging the advantages of convolutional neural networks in image feature extraction and utilizing vast radar detection data to design an implementation of weather radar echo extrapolation models. This strategy aims to improve prediction accuracy with a lower number of network parameters and network complexity. (3) Developing an optimization method for short-term extrapolation of radar echo images based on generative adversarial network models. By introducing the concept of adversarial training, using the radar echo image short-term extrapolation model as the generator and multilayer convolutional neural networks as the discriminator, different optimization objectives are set to enhance the prediction accuracy of the extrapolation model for high-intensity echo areas of radar images.

### 4.2. Developing All-Weather Process Observation Techniques for Weather Radar (Keywords in Table 7: Scanning Strategy, Observation Mode)

The all-weather process monitoring technology of weather radar is a reflection of whether the radar can achieve comprehensive, accurate, and complete monitoring capabilities for weather processes [82]. The all-weather process mainly consists of four stages: the clear sky atmospheric stage, cloud formation stage, precipitation stage, and meteorological disaster stage, with each stage closely related to the others. However, to effectively monitor the four different stages of weather processes, it is not only necessary to have corresponding technologies tailored to the characteristics of each stage in radar hardware but more importantly, to establish appropriate scanning strategies and effective observation modes based on the characteristics of each stage. Only when a radar unit is equipped with the best scanning strategy and observation mode can it fully utilize the monitoring capabilities that range from the clear-sky atmospheric state, through various clouds and precipitation, to the formation of disasters.

However, as an essential component of weather radar, the design process of scanning strategies and observation modes must clearly recognize the following: under the Doppler pulse technology radar system, the maximum unambiguous range and the maximum unambiguous velocity are a contradictory pair; fast sampling conflicts with obtaining high-precision data; high spatial resolution vertical fine structure sampling conflicts with high time resolution; and using oversampling techniques to improve azimuthal spatial resolution conflicts with sampling precision and data quality. Effectively resolving these contradictions and finding the optimal balance point is the key to advancing all-weather process observation techniques for weather radar.

### 4.3. The Refinement Upgrade of Weather Radar (Keywords in Table 7: Upgrade Radar Images, Fine Feature Extraction, Refined Detection)

With the continuous advancement and development of electronic technology, computer technology, and meteorological science, disaster prevention and mitigation have placed higher demands on weather radar technology. Currently, weather radar technology is generally advancing towards a deeper understanding of both macroscopic and microscopic physical characteristics, high spatiotemporal resolution, and more precise quantitative techniques [83,84]. Technologies from other industries are being continuously applied in the field of meteorological detection and realized through weather radar. For example, phased array technology and pulse compression technology have already been applied in weather radar to enhance the radar’s temporal and spatial resolution, aiming to improve the understanding of atmospheric and various weather system structures. Similarly, dual-polarization technology has been widely utilized in weather radar to advance the understanding of microphysical properties in the atmosphere. Additionally, technologies such as dual-radar observation, multisite radar technology, phase-coding technology, and continuous wave radar technology, among others, are progressively being applied in weather radar. Exploring how to further enhance the detection accuracy, sampling resolution (temporal and spatial resolution), and hydrometeor identification capability of weather radar using these new technologies is also an important future research direction in the field of weather radar.

**Figure 11:**
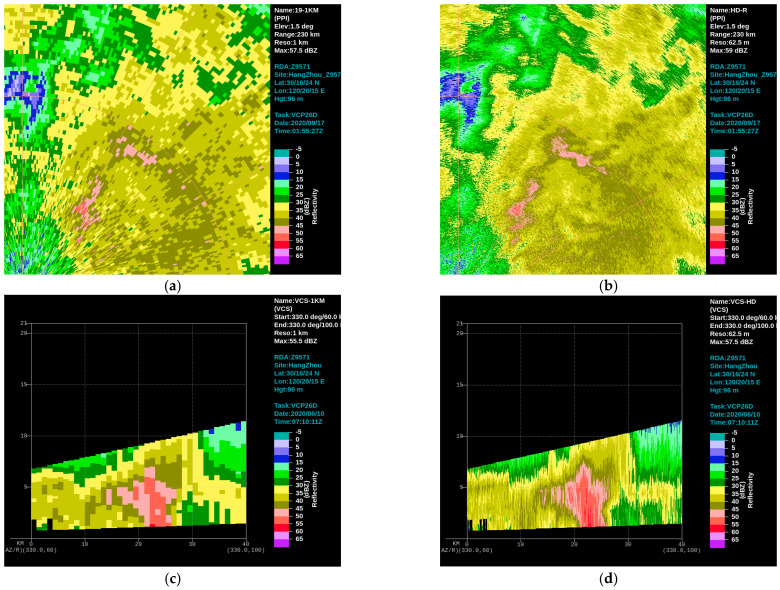
The contrast of reflectivity before (**a**,**c**) and after (**b**,**d**) rapid and refined technological upgrades (horizontal perspective: (**a**,**b**); profile perspective: (**c**,**d**)).

It is worth mentioning that China is currently undergoing rapid and refined technological upgrades to the national next-generation weather radar network. The goal is to enhance the distance resolution of detection pulses, accelerate the speed of weather radar scanning, and comprehensively improve the spatiotemporal resolution of detection data without compromising data quality. With the adoption of rapid and refined technology, the volume scanning cycle of weather radar can be shortened from the current 6 min to 3–4 min, and the data resolution can be enhanced to a refined level of 0.5° × 62.5 m from 1° × 250 m. Rapid and refined detection technology directly increases the amount of radar echo data from the source of data collection, making positive contributions to the analysis of characteristics of medium- and small-scale weather, radar-based quantitative precipitation estimation, and related operational applications (Figure 11).

### 4.4. Developing Networked Collaborative Observation Technology for Weather Radars (Keywords in Table 7: Networked Radars, Collaborative Observation)

For a weather radar network, networked radars may consist of different types of weather radars or multiple radars of the same type to achieve effective detection over a larger area [85]. However, in the context of collaborative observation with weather radars, emphasis should be placed on coordinating observations from various types of radars to enable multiple-frequency and multicategory weather radars to effectively and comprehensively detect targets in the same space at the same time [86]. This facilitates a comprehensive understanding of the atmosphere, enabling intercomparison and validation of different types of radars. Collaborative observation technology for weather radars is a crucial direction for weather radar technological advancement. Currently, collaborative observation technology is progressing in two main directions: one is comprehensive observation technology through coordinated networking, which involves integrating various radars through observation and quality control methods; the other involves utilizing multifrequency detection devices to achieve integrated collaborative observations on the same platform. Regardless of the specific collaborative observation technology, their ultimate goal is to achieve synchronous observations in time and space, leading to mutual comparison and validation, thereby generating fused products of various physical quantities to comprehensively reveal atmospheric characteristics.

For weather radar, in-depth research into data fusion algorithms among different frequency bands of weather radar and between weather radar and other types of observations can provide us with data products that are more physically meaningful and have greater practical application capabilities. For instance, utilizing multifrequency radars (S-band, X-band, and Ka-band) can offer characteristic distributions of particle size spectra ranging from large raindrops and small raindrops to cloud droplets, thereby providing a particle spectrum distribution that better approximates the actual composition of precipitation cloud systems. Similarly, combining reflectivity data obtained from weather radar with ground-based rain gauges for precipitation estimation not only yields feature information that better aligns with the actual precipitation intensity structure but also provides higher-resolution precipitation distribution characteristics. Furthermore, merging Velocity Azimuth Display (VAD) wind field information obtained from weather radar with wind field information from L-band wind profilers can reveal detailed structures of mid-to-low atmospheric wind fields, thereby compensating for the significant deficiencies in large-scale sounding data.

## 5. Conclusions

The main goal of this research is to explore the current status and evolving trends in weather radar research from 1945 to 2024. To accomplish this, a detailed analysis using scientometric methods is carried out from four perspectives: general features, collaboration, co-citation, and keyword co-occurrence. By analyzing a dataset of 13,981 publications from the WoS core collection database, this study seeks to unveil and visually depict, for the first time, the fundamental conceptual structures that have shaped the knowledge domain of weather radar over an 80-year period. Additionally, the research delves into the future pathways and advancements anticipated in weather radar research. In conclusion, this study identifies the following key insights:

(1) The number of publications in weather radar research was notably low between 1945 and 1990, suggesting a period of stagnation. However, since 1990, there has been a significant and continuous increase in both publications and citations, peaking in 2022 with 1083 publications and 13,832 citations, reflecting sustained growth and interest in this field of study.
(2) The United States, China, and European countries have played a highly influential role in the swift advancement of weather radar research, with enthusiastic participation from institutions and authors in each country. International collaboration among authors, institutions, and countries is currently the prevailing trend and has contributed significantly to the rapid growth of the weather radar research area over the past 80 years.
(3) A total of 30 distinct co-citation clusters have been uncovered, outlining the knowledge structure in weather radar research. The co-citation analyses of author and journal reveal a continuous and steady advancement in the field from 1945 to 2024. Notably, deep learning emerges as the most dynamic cluster in contemporary weather radar research, attracting considerable attention and producing substantial results.
(4) During the last 80 years, the investigative emphasis in the field of weather radar has gradually shifted from the research and improvement of weather radar hardware and software and the initial applications of observational products to the integration of AI technologies and multifunctional applications across multiple scenarios.
(5) Drawing from the preceding analysis, this study outlines four key areas for future research in the field of weather radar: the application of AI technology, the development of all-weather process observation techniques, the refinement upgrade, and the development of networked collaborative observation technology.

In a practical sense, the goal of this study is to provide substantial support to scholars deeply engaged in weather radar research by enhancing their understanding of its evolution. The findings from this study can serve as a useful guidebook, helping researchers quickly locate pertinent publications for reference and suitable journals for submitting articles. Furthermore, policymakers can utilize this comprehensive review as a solid foundation for decision-making. Nevertheless, there is potential for additional enhancements in this study. For example, the bibliography for this study includes academic papers sourced from the WoS core collection database. Although WoS is recognized as a reliable study resource, broadening the spectrum of data sources in upcoming academic endeavors could possibly enrich the precision of knowledge frameworks within the domain of weather radar. Furthermore, scientometric mapping represents a data-centric and impartial technique for studying knowledge spheres, aiming to mitigate subjective biases. However, interpreting mapping outcomes effectively requires integrating expert perspectives and domain-specific expertise. In future research endeavors, involving external specialists to critically examine the findings could offer a more logical approach.

## Figures and Tables

**Table 1:** The leading 10 authors based on publication count.

Author	Publications	Centrality	Year	Author	Publications	Centrality	Year
Chandrasekar V.	118	0.13	1992	Wang Xuguang	39	0.02	2017
Xue Ming	95	0.06	2007	Horton Kyle G	37	0.02	2015
Zhang Guifu	60	0.06	2008	Berne Alexis	34	0.03	2010
Chen Haonan	48	0.05	2015	Ushio Tomoo	31	0.02	2015
Gao Jidong	45	0.03	2010	Sun Juanzhen	29	0.03	2008

**Table 2:** The leading 10 institutions based on publication count.

Institution	Publications	Year (First Publication)	Country
National Oceanic Atmospheric Admin (NOAA)	1252	1975	USA
University of Oklahoma System	1032	1983	USA
University of Oklahoma—Norman	892	1983	USA
Chinese Academy of Sciences	563	1993	China
National Aeronautics & Space Administration (NASA)	511	1991	USA
China Meteorological Administration	486	2000	China
National Center Atmospheric Research (NCAR)	453	1983	USA
Colorado State University	358	1989	USA
Nanjing University of Information Science & Technology	347	2008	China
Centre National de la Recherche Scientifique (CNRS)	332	1991	France

**Table 3:** The leading 10 countries based on publication count.

Country	Publications	Centrality	Country	Publications	Centrality
USA	4926	0.13	France	635	0.23
China	2893	0.04	India	592	0.03
Germany	923	0.16	Canada	575	0.12
England	895	0.26	Japan	473	0.07
Italy	663	0.14	Switzerland	367	0.05

**Table 4:** Overview of the top 18 clusters (exclude irrelevant clusters).

Cluster ID	Size	Silhouette	Average Year	Label
0	189	0.892	2010	Ensemble forecast
1	178	0.938	2004	Cloud analysis
2	147	0.949	1996	To-ground lightning
3	135	0.915	2017	On-forecast system
4	126	0.925	2006	Hydrological model
5	109	0.917	2011	Adaptive habit model
6	90	0.933	2015	Urban hydrology
7	84	0.942	2016	Rainfall product
8	80	0.972	1991	Severe weather
9	78	0.968	1998	Differential reflectivity
10	72	0.967	2019	Precipitation nowcasting
11	69	0.982	2016	Migrating bird
14	55	0.948	1995	National Severe Storms Laboratory tornado detection
15	49	0.964	2014	Alpine region
16	45	0.982	1999	TRMM precipitation radar
17	43	0.966	2008	Precipitation measurement
18	40	0.983	1995	Structure evolution
19	38	0.928	2019	South China

**Table 5:** The leading 10 authors based on co-citation frequency, centrality, and burst strength.

Co-Citation Frequency	Centrality	Burst Strength
Doviak R. J. (956)	Atlas D. (0.13)	Doviak R. J. (90.23)
Bringi V. N. (858)	Browning K. A. (0.09)	Hersbach H. (79.56)
Skamarock W. C. (832)	Battan L. J. (0.06)	Joss J. (76.82)
Houze R. A. (764)	Wilson J. W. (0.06)	Collier C. G. (68.28)
Zrnic D. S. (758)	Marshall J. S. (0.06)	Wang Y. (56.36)
Wilson J. W. (729)	Sun J. Z. (0.06)	Browning K. A. (55.24)
Marshall J. S. (686)	Crum T. D. (0.06)	Kitchen M. (53.86)
Ryzhkov A. V. (640)	Collier C. G. (0.06)	He K. M. (49.83)
Kain J. S. (626)	Doviak R. J. (0.06)	Ronneberger O. (48.92)
Hong S. Y. (613)	Germann U. (0.06)	Fulton R. A. (47.26)

**Table 6:** The leading 10 journals based on co-citation frequency, centrality, and burst strength.

Co-Citation Frequency	Centrality	Burst Strength
*B. Am. Meteorol. Soc. (5027)*	*Nature (0.06)*	*Remote Sens.-Basel (240.22)*
*Mon. Weather Rev. (4768)*	*J. Meteorol. (0.05)*	*J. Appl. Meteorol. (190.81)*
*J. Atmos. Ocean. Tech. (4506)*	*Science (0.05)*	*Thesis (163.36)*
*J. Appl. Meteorol. (4418)*	*B. Am. Meteorol. Soc. (0.04)*	*Atmosphere-Basel (125.23)*
*J. Atmos. Sci*. *(3992)*	*Adv. Geophys. (0.04)*	*Arxiv (120.47)*
*J. Geophys. Res.-Atmos. (3889)*	*17 C. Rad. Met. Seattl. (0.04)*	*J. Atmos. Sci. (118.24)*
*Wea. Forecasting (3811)*	*27 C. Rad. Met. Vail. Co. (0.04)*	*J. Clim. Appl. Meteorol. (111.78)*
*Q. J. Roy. Meteor. Soc. (3750)*	*P. IEEE (0.04)*	*Proc. Cvpr. IEEE (108.92)*
*IEEE T. Geosci. Remote (3683)*	*IEEE T. Antenn. Propag. (0.03)*	*J. Atmos. Ocean. Tech. (102.15)*
*Geophys. Res. Lett*. *(3384)*	*Auk. (0.03)*	*Doppler Radar Weathe (97.69)*

**Table 7:** The top 25 keywords exhibiting the most pronounced citation bursts (excluding irrelevant keywords).

Keywords	Year	Strength	Begin	End	1945–2024
Doppler radar	1991	29.53	1991	2011	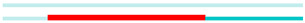
Mesoscale	1992	16.22	1992	2012	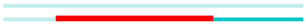
Boundary layer	1993	13.67	1993	2008	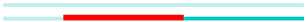
Fields	1996	21.82	1996	2010	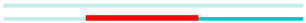
WSR 88D	1997	43.85	1997	2014	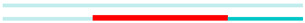
Sensitivity	1999	13.27	1999	2007	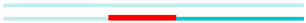
Range	2000	18.12	2000	2008	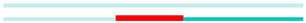
Reflectivity	1986	17.64	2000	2013	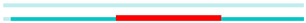
Retrieval	1997	13.62	2001	2013	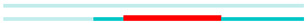
Propagation	2003	11.85	2003	2012	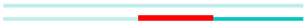
Rainfall	1990	15.67	2004	2009	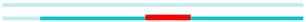
Hydrology	2005	13.68	2005	2015	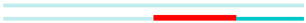
Shape	2005	12.43	2005	2012	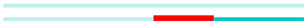
Machine learning	2019	27.63	2020	2024	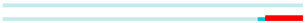
Upgrade radar images	2020	15.24	2020	2024	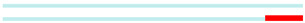
Numerical weather prediction/forecasting	2020	13.43	2020	2024	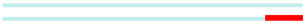
Deep learning	2019	52.86	2021	2024	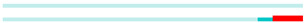
Laser radar	1998	15.83	2021	2024	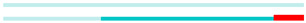
Convolutional neural network	2021	14.64	2021	2024	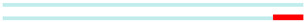
Scanning strategy	2021	11.42	2021	2024	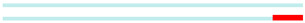
Fine feature extraction	2020	10.73	2021	2024	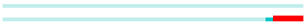
Networked radars	2012	15.76	2022	2024	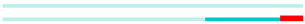
Observation mode	2022	13.84	2022	2024	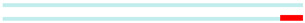
Refined detection	2022	12.15	2022	2024	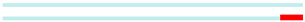
Collaborative observation	2019	11.56	2022	2024	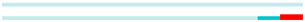

## Data Availability

The data are available upon reasonable request from the author of the paper.

## Appendix A. The Commonly Used Terms and Their Meanings in Scientometric Mapping

When analyzing a network visualization generated by CiteSpace, there are several key visual patterns and elements to pay special attention to:

### Appendix A.1. Node Size

The size of a node usually indicates its importance or prominence in the network. Larger nodes might represent highly cited papers, influential researchers, or key concepts.

### Appendix A.2. Node Color and Tree Rings

The color of nodes and the presence of tree rings (especially red rings) can be significant. The color often represents the time frame of the publication or the emergence of a concept. Red tree rings indicate citation bursts, suggesting that the work has received increasing attention over a specific period.

### Appendix A.3. Links between Nodes

The connections or links between nodes show relationships, such as co-authorship, co-citation, or thematic similarity. The strength and number of these links can indicate the degree of association.

### Appendix A.4. Clusters

Clusters are groups of closely related nodes. They often represent a specific theme or topic within the broader research area. Larger clusters typically indicate more research activity or interest in that particular area.

### Appendix A.5. Betweenness Centrality

The betweenness centrality is defined for each node in a network. It measures how likely an arbitrary shortest path in the network will go through the node. A node with a high betweenness centrality is likely to sit in the middle of two large communities, or sub-networks, hence the name betweenness.

In CiteSpace, a node with a high betweenness centrality is shown with a purple ring. The thickness of the purple ring depicts the value of the betweenness centrality.

The use of betweenness centrality in CiteSpace is guided by the structural hole theory. The theory was originally developed for social networks. An insightful observation is that the connectivity, or the lack of it, can guide us to find the most valuable nodes in a network. CiteSpace builds on top of these theories to detect boundary-spanning potentials and novel brokerage connections in scholarly publications.

### Appendix A.6. Circular View

The “Circular View” in CiteSpace is a visualization method for displaying the structure of citation networks. In this view, the citation network is presented as a circle, where each node on the circumference represents a document or author, and the position of the nodes reflects their relevance or similarity within the network. The lines connecting the nodes represent citation relationships or collaborations between documents or authors.

The main feature of the Circular View is the grouping of nodes based on their similarity or relevance, arranged along the circumference according to certain rules. This arrangement allows users to intuitively observe the relationships and interactions between different topics or fields, facilitating a better understanding of the structure and content of the citation network.

Through the Circular View, researchers can identify thematic groups or research areas within the network, explore their internal structures and connections, and further analyze key nodes or pivotal documents to delve into the academic development and dynamic changes within specific fields.

### Appendix A.7. Timezone View

The “Timezone View” in CiteSpace refers to the visualization of academic activities over different time periods within a citation network. This feature helps researchers understand the evolving trends and changes in research within a specific field over time.

In the Timezone View, the citation network is divided into different time intervals based on the publication years of the citations. Each time interval consists of nodes representing literature or authors with similar themes or relevance, with node size typically indicating their influence or citation count. The edges between nodes represent citation relationships or collaboration.

Through the Timezone View, researchers can observe the research hotspots, changes in academic collaboration, and development trends within a specific field over different time periods. This enables them to better understand the historical development trajectory of the field and guide future research directions and collaboration strategies.

### Appendix A.8. Co-Citations and Co-Occurrences

Co-citations refer to the fact that two references are cited by the third article. Traditionally as long as two references are cited anywhere within the third article, they are considered as co-cited. If the full text of the third article is available, obviously one can narrow down the scope to sections, paragraphs, or sentences.

Citing a reference may serve many purposes and may be motivated by a wide variety of reasons. However, the way a reference has been cited may function similarly to reference an underlying concept.

An instance of a co-citation is a local event. It only involves two references. These local events contribute to the formation of a network of co-cited references. A primary goal of CiteSpace is to analyze such networks so as to identify patterns and trends concerning the structure and dynamics of the underlying scientific literature.

A co-citation connection between references R1 and R2 is depicted in a visualized network based on the similarity between the two references involved. Assume our dataset consists of 10,000 articles. R1 is cited by 100 of the 10,000 articles and R2 is cited by 100 of them. The similarity is computed based on the articles that cited both R1 and R2 out of the two sets of citing articles. Suppose the number of articles that cited both R1 and R2 is 30, divided by the square root of the multiplication of 100 and 100, which is 100, then the similarity, or the weight of the co-citation link, is 0.30. The maximum similarity of 1.00 is achieved when the two 100-article sets overlap completely, whereas the minimum similarity of 0.00 corresponds to the situation of no overlapping citing articles, i.e., R1 and R2 are never co-cited by any of the 10,000 articles. CiteSpace provides several ways to measure the similarity.

Co-citations can be seen as a special case of co-occurrences, which can include co-occurring words, or co-words, and another types of entities such as co-authors, or author co-citations.

### Appendix A.9. Silhouette Scores

The silhouette score of a cluster measures the homogeneity of the cluster, which answers the question of whether cluster members are lumped together based on what they have in common in some aspects. In other words, a cluster with a high silhouette score is considered more meaningful than a grouping with a low silhouette score.

### Appendix A.10. Burst Detection

A burst refers to a frequency surge of a particular type of events, for example, a surge of citations to a Nobel Prize winning publication.

CiteSpace supports burst detection on several types of events: (1) single- or multiword phrases from the title, abstract, or other parts of a publication; (2) the number of citation counts of cited references over time; (3) the frequencies of keyword appearances over time; and (4) the number of publications by an author, an institution, or a country.

In CiteSpace, users may adjust the burst detection parameters in the Burstness tab in the Control Panel. For example, to find more items of burst, i.e., increase the sensitivity of the burst detection, reduce the gamma value. To reduce the number of items of burst to be identified, increase the minimum duration.

### Appendix A.11. Timeline View

The “Timeline View” in CiteSpace is a visualization tool designed to illustrate the evolving scholarly activities within citation networks over time. In this view, the citation network is represented along a timeline, where nodes at different time points represent documents or authors, with node size typically indicating their influence or citation count.

The Timeline View aids researchers in understanding the research trends and evolution within a particular field by showcasing scholarly activities over different time periods. Users can select specific time intervals on the timeline to observe research focuses, author activities, citation patterns, and more within that timeframe.

Through the Timeline View, researchers can track the developmental trajectory of a specific field, identify shifts in research hotspots, and observe changes in academic collaboration patterns. This enables them to gain a comprehensive understanding of the dynamic changes within the field, guiding future research directions and collaboration strategies.

### Appendix A.12. Landscape View

In CiteSpace, the “Landscape View” is a method used to visually represent the relationships between different documents or authors within a citation network. In the Landscape View, the citation network is presented as a two-dimensional plane, where each node represents a document or author, and the position of the nodes reflects their similarity or association within the network.

The key feature of the Landscape View is the layout of nodes based on their similarity, positioning similar nodes closer to each other. This layout allows users to intuitively observe relationships between different themes or fields within the network, as well as the group structures and positions of key nodes within the network.

Through the Landscape View, researchers can discover different research groups or domains within the citation network and explore their relationships and interactions. This helps them understand the dynamics of research within specific fields, identify key documents or authors in related areas, and further delve into the study and analysis of specific topics or domains.
